# Coronavirus disease 2019 (COVID-19) impact on central-line-associated bloodstream infections (CLABSI): a systematic review

**DOI:** 10.1016/j.infpip.2023.100313

**Published:** 2023-10-13

**Authors:** Giovanni Satta, Timothy M. Rawson, Luke S.P. Moore

**Affiliations:** a Department of Infection, University College London Hospitals NHS Foundation Trust, London, UK; b Centre for Antimicrobial Optimisation, Imperial College London, London, UK; c Clinical Infection Department, Chelsea and Westminster NHS Foundation Trust, London, UK

**Keywords:** Central line-associated bloodstream infections, CLABSI, COVID-19 infection, Global epidemiology

## Abstract

**Introduction:**

Central line-associated bloodstream infections (CLABSI) are an important clinical and public health issue, impacted by the purported increase in healthcare-associated infections (including CLABSI) during the COVID-19 pandemic. This review evaluates the impact of COVID-19 on CLABSI at a global level, to determine risk factors, effective preventive measures and microbiological epidemiology.

**Methods:**

A systematic literature review was performed using a PECO framework, with COVID-19 infection as the exposure measure and CLABSI rates as the main outcome of interest, pre- and during the pandemic.

**Results:**

Overall, most studies (17 of *N=*21) found a significant increase in CLABSI incidence/rates during the pandemic. Four studies showed a reduction (*N=*1) or no increase (*N*=3). High workload, redeployment, and ‘overwhelmed’ healthcare staff were recurrent risk-factor themes, likely to have negatively influenced basic infection control practices, including compliance with hand hygiene and line care bundles. Microbiological epidemiology was also impacted, with an increase in enterococcal infections and other pathogens.

**Conclusion:**

The COVID-19 pandemic significantly impacted CLABSI incidence/rates. Observations from the different studies highlight significant gaps in healthcare associated infections (HCAI) knowledge and practice during the pandemic, and the importance of identifying preventive measures effective in reducing CLABSI, essential to health system resilience for future pandemics. Central to this are changes to CLABSI surveillance, as reporting is not mandatory in many healthcare systems. An audit tool combined with regular assessments of the compliance with infection control measures and line care bundles also remains an essential step in the prevention of CLABSI.

## Introduction

A central-line-associated bloodstream infection (CLABSI) is defined by the Centers for Disease Control (CDC) as the isolation of a pathogen from a blood culture (a single blood culture for organism not commonly present on the skin, and two or more blood cultures for organism commonly present on the skin) in a patient who had an intravenous central line at the time of infection or within 48 hours before development of infection. The source cannot be related to any other infection the patient might have at any other site and must not have been present when the patient was admitted to the healthcare facility [1].

Additional terms are also in use, with different defining criteria when compared to the CDC definition. Those include bloodstream infections (BSI), catheter-related bloodstream infection or CRBSI (based on the Infectious Diseases Society of America guidelines, currently being updated) [2] and catheter-associated bloodstream infection or CABSI (based on the ICCQIP – Infection in Critical Care Quality Improvement Programme definition in Europe) [3]. Of note, CRBSI may require a more definitive diagnosis (potentially not available in all hospitals) and in CABSI the denominator may be different, using admission days versus line days. This review will focus on CLABSI/CRBSI/CABSI, but not the wider BSI. The term CLABSI will be used throughout, unless specified otherwise.

CLABSI are an important public health issue as they have a significant impact on patients' morbidity and mortality and increase health care costs and length of hospital admissions [4]. Among all the healthcare-associated infections, the costs caused by a CLABSI amount to approximately $46,000 per case, with most infections preventable with proper aseptic techniques, surveillance, and care bundles [5]. An estimated 250,000 bloodstream infections occur annually, and most are related to the presence of intravascular devices. In the United States (US), the CLABSI rate in intensive care units (ICUs) is estimated to be 0.8 per 1000 central line days, but international data from 50 different countries reported a much higher CLABSI rate of 44.6 per 1000 central line days [6].

A recent analysis conducted by the CDC has revealed a continued increase in healthcare-associated infections (HCAI) in US hospitals during 2021, the second year of the COVID-19 pandemic [7]. This is also in line with reports from other countries, with significant variation in traditional epidemiology of bloodstream infections identified during the COVID-19 pandemic, including higher rates of bacterial infections and diversity of microbial pathogens [8,9]. However, there is still variation in some of the reporting and the increase in CLABSI rates has not been consistent in all healthcare institutions, highlighting other factors, such as compliance with infection control measures and local line care policies, may have had an impact on the overall epidemiology.

Microbiologically, Gram-positive bacteria (coagulase-negative staphylococci, enterococci, and *Staphylococcus aureus*) are the most common causative organisms of CLABSI, followed by Gram-negative organisms (such as, *Klebsiella* spp., *Enterobacter* spp., *Pseudomonas* spp.), fungal/*Candida* spp. (11.8%), and others (10.5%) [1]. During the COVID-19 pandemic, the National Healthcare Safety Network (NHSN) data also showed an increase in the proportion of pathogens identified as *Enterococcus faecalis* and coagulase-negative staphylococci during 2020 when compared to 2019 [10].

This review interrogated extant medical literature on the epidemiology of CLABSI during the COVID-19 pandemic to assess the magnitude of the problem at a global level. A secondary aim was to determine if any impact in CLABSI incidence was associated with identifiable risk factors (i.e., patient's comorbidities, type of hospital ward and/or non-compliance with infection control practices) and if any preventive measures demonstrated an effect in reducing the incidence of CLABSI during the pandemic. Finally, a tertiary aim was to review the microbiological epidemiology and if the changes in microbial pathogens were observed worldwide.

## Methods

A systematic literature review was performed using an online tool for evidence synthesis (Covidence; Australia). The MEDLINE and EMBASE databases were searched for articles from 1st January 2020 to the 1^st^ July 2023 using a combination of broad-based search criteria including COVID-19, coronavirus, CLABSI, central-line-associated, healthcare-associated, bloodstream infections. Only articles in English were included.

Two independent reviewers (GS & TMR) screened the initial articles through their abstracts and selected the papers for full extraction and reading, based on a PECO (Population, Exposure, Comparison, Outcome) framework. COVID-19 infection was considered as the exposure measure of interest, whilst the CLABSI rates was the main outcome measure of interest, comparing rates pre- and during the pandemic. Country, type of study (prospective versus retrospective cohort studies, case series, etc), including the study dataset, were all included in the final comparison, as well as the principal findings (related to CLABSI).

Additional opinion articles and commentaries were also screened, in particular looking for preventive measures that have been demonstrated to be effective in reducing the risk of CLABSI during the recent pandemic. As an existing robust literature on reducing CLABSI in general (but not related to COVID-19) is already present and spans over the last two decades and to keep the search more focused, only articles specifically related to COVID-19 and CLABSI were considered.

### Risk of bias

Risk of bias for individual studies were assessed in line with Cochrane recommendations. For non-randomised studies the Risk of Bias in Non-randomised studies of Evaluations (ROBINS-E) assessment tool was used [11]. Risk of bias was assessed by two reviewers (TMR & LSPM) independently of each other. Studies were evaluated against specific risk of bias domains with the risk of individual domains used to determine an overall risk of bias for the study. Each study was ranked as low, some, high, or very high risk of bias overall. Where disagreement in domain scoring occurred, a third reviewer (GS) assessed the study and differences were discussed to reach consensus.

## Results

**Figure 1:**
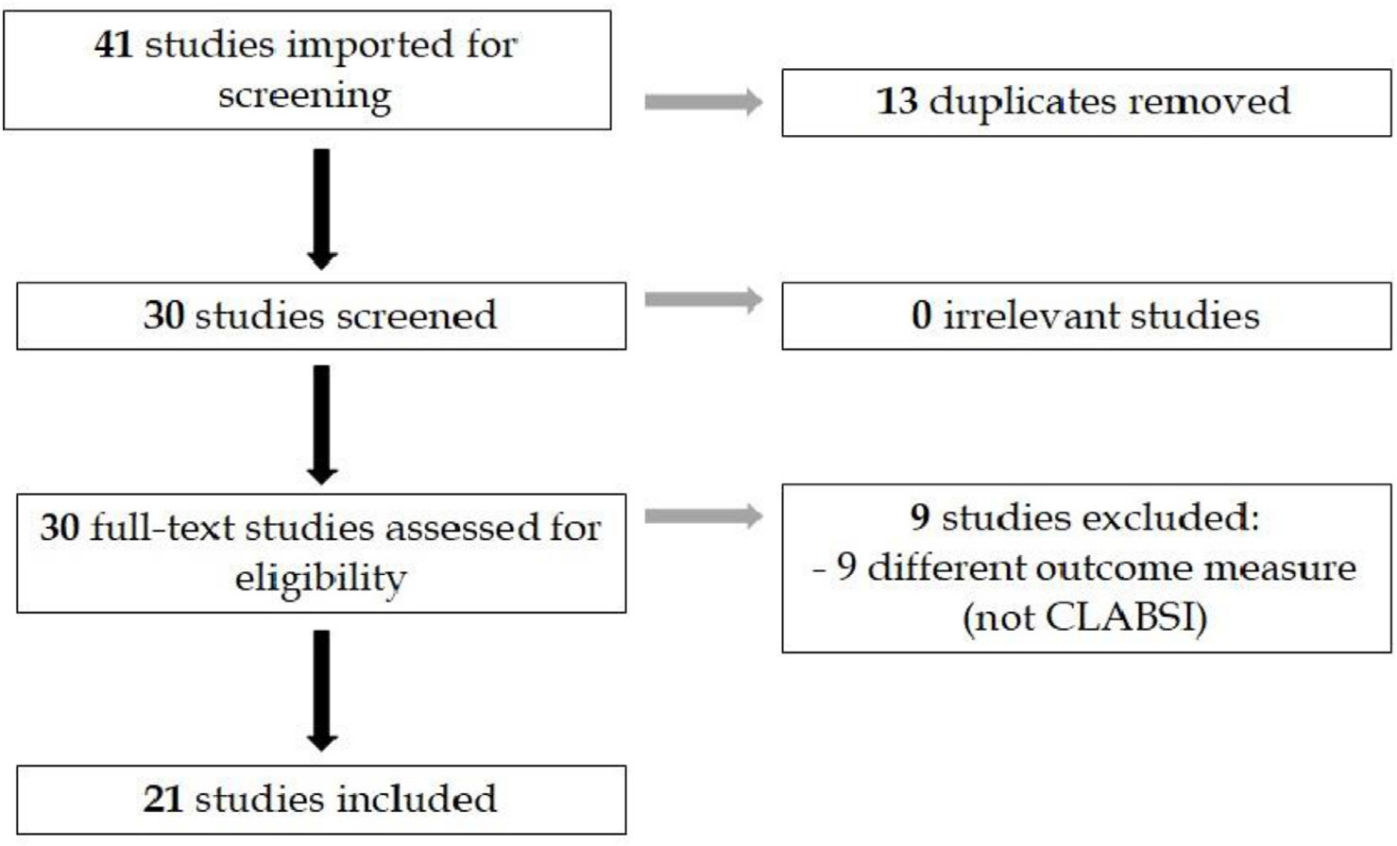
PRISMA flowchart, summarizing initial number of papers imported, screened and included in the final review. Nine studies were excluded as they did not include CLABSI rates as their main outcome measure.

The initial search identified 43 studies on the topic of CLABSI and COVID-19. The Covidence systematic review software eliminated 13 duplicate papers, reducing the number of articles to be screened to 30 (Figure 1). The search did not find any existing narrative or systematic reviews on CLABSI and COVID-19 or clinical trials on the topic.

All 30 articles were reviewed, 21 of which included original data on CLABSI rates and were included in the literature review, whilst all other studies (*N*=9) did not include the CLABSI rate as the main outcome measure of interest (Table I, list of excluded studies). In terms of location, twelve of the studies included in the literature review were performed in USA, one in Brazil, two in Europe (Germany and the Netherlands), three in Saudi Arabia, one in South Korea, one in India and one in multiple low- and middle-income countries (LMICs). Two studies from USA and from the same research group [7,12] have used the same dataset but at different time points as the pandemic evolved, but there were still considered as separate for the purpose of this report. A list of all studies reporting CLABSI rates (*N*=21) and their main findings are summarized in Table II.

**Table I:** List of excluded studies (alphabetical order) and further explanation for exclusion

List of excluded studies	Reason
Afzal *et al.*, 2022 [42]	BSI was the main outcome measure, no CLABSI rates were reported
Cataldo *et al.*, 2020 [9]	BSI was the main outcome measure, no CLABSI rates were reported
Denny *et al.*, 2021 [48]	No CLABSI rates were reported
Giacobbe *et al.*, 2020 [55]	BSI was the main outcome measure, no CLABSI rates were reported
Giacobbe *et al.*, 2021 [45]	No CLABSI rates were reported
McAlearney *et al.*, 2021 [56]	Qualitative study, no CLABSI rates were reported
Najjar-Debbiny *et al.*, 2022 [40]	National survey with no CLABSI rates reported
Ripa *et al.*, 2021 [57]	No CLABSI rates were reported
Thaprawat *et al.*, 2022 [37]	National survey with no CLABSI rates reported

**Table II:** Summary of the studies included (*N*=16) in the literature review. The main information are categorized in different columns to include: outcome measure(s) of interest (mention to other HCAI have been included for completeness), country where the study was conducted, type of study, study dataset (including number of hospitals or patients if available) and brief summary of the principal findings (related to CLABSI)

Article's citation	Exposure measure of interest	Outcome measure(s) of interest	Country	Type of study	Study dataset	Principal findings (related to CLABSI)	Notes
Advani *et al.*, 2022 [19]	COVID-19	Healthcare associated infections (HAI), including central-line-associated bloodstream infections (CLABSI), catheter-associated urinary tract infections (CAUTI), *Clostridioides difficile* infections (CDI), and ventilator-associated events (VAE)	USA	Retrospective longitudinal multicenter cohort study	53 hospitals in Southeastern United States	CLABSI increased by 24%, during the pandemic period On stratifying the analysis by hospital characteristics, the impact of the pandemic on HCAIs was more significant in smaller community hospitals	**Increase**
AlAhdal *et al.*, 2022 [15]	COVID-19	Device associated infections (DAI), compliance with hand hygiene and other prevention bundles in ICU	Saudi Arabia	Retrospective observational study	Single 500-bed hospital, including 80 adult ICU beds	There was no significant difference in the number of device associated infections or compliance with hand hygiene and other bundles	**No difference**
Alsaffar [32]	COVID-19	CLABSI and CAUTI data	Saudi Arabia	Retrospective data analysis	Data from the Saudi Health Electronic Surveillance Network (HESN) covering Ministry of Health Hospitals	The COVID-19 pandemic was associated with increased CLABSI rates	**Increase**
Al-Tawfig [16]	COVID-19	VAE, CAUTI and CLABSI data	Saudi Arabia	Retrospective data analysis	Hospital network with 5 ICUs	No significant difference observed for CLABSI (but limited to 2020 only)	**No difference**
Baker *et al.*, 2022 [17]	COVID-19	CLABSI, CAUTI, methicillin-resistant *Staphylococcus aureus* (MRSA) bacteremias, and *Clostridioides difficile* infections (CDI)	USA	Prospective cohort study	148 hospitals affiliated with the Health Corporation of America (HCA)	CLABSI and MRSA bacteraemias increased during the COVID pandemic	**Increase**
Ben-Aderet *et al.*, 2022 [22]	COVID-19	CLABSI rates	USA	Retrospective cohort study	Academic 889-bed tertiary-care teaching hospital Los Angeles	The CLABSI rate during COVID-19 was significantly higher than non–COVID-19 period	**Increase**
Evans *et al.*, 2022 [24]	COVID-19	HAIs, including CLABSI, VAE, CAUTI, CDI and methicillin-resistant MRSA infections	USA	Retrospective data analysis	128 acute-care and 132 long-term care Veterans Affairs (VA) facilities	During the pandemic, the average monthly CLABSI rates increased significantly by 31% with increased catheter utilization ratios	**Increase**
Fakih *et al.*, 2022 [8]	COVID-19	CLABSI and CAUTI rates	USA	Retrospective data analysis	78 hospitals from a single healthcare system all over USA (Ascension)	CLABSI rates increased by 51.0% during the pandemic period from 0.56 to 0.85 per 1000-line days (*P<*0.001) and by 62.9% from 1.00 to 1.64 per 10,000 patient days (*P<*0.001)	**Increase**
Geffers *et al.*, 2022 [14]	COVID-19	CLABSI, CAUTI, ventilator-associated lower respiratory infections (VALRTI) and bloodstream infections associated with the use of Extracorporeal-Life-Support-Systems (ECLSABSI)	Germany	Retrospective data analysis	National Reference Center for Surveillance of Nosocomial Infections (921 German ICUs)	No increase was shown for CLABSI	**No difference** The lack of HAI increase in German ICUs may be due to the lower overall incidence of COVID-19 in Germany in 2020 compared with US or the very high availability of ICU beds
Halverson *et al.*, 2022 [20]	COVID-19	HAIs, including CLABSI, CAUTI, CDI and MRSA infections	USA	Retrospective cohort study	2 hospitals in Illinois, 159 bed community and 894 bed academic hospital	Significant increase in CLABSI per 1000 patient days and 1000 device days during the pandemic	**Increase**
Lee [30]	COVID-19	BSI, CLABSI, CAUTI and VAP	South Korea	Retrospective data analysis	Data from the Korean National Healthcare-Associated Infections Surveillance System	The rates of BSI and CLABSI significantly increased during the COVID-19 pandemic compared to the pre-COVID-19 period in large-sized hospitals, whereas these rates significantly decreased in small-to-medium-sized hospitals	**Increase**
Meynaar *et al.*, 2022 [29]	COVID-19	CLABSI rates and use of dexamethasone and interleukin antagonists	Netherlands	Retrospective data analysis	Intensive Care Unit at the Haga Teaching Hospital (The Hague)	The risk of CLABSI was significantly increased among COVID-19 patients treated with dexamethasone	**Increase**
Mitra *et al.*, 2021 [13]	COVID-19	CAUTI, CLABSI, ventilator-associated pneumonia (VAP), surgical site infections (SSIs) and hand hygiene compliance rates	India	Retrospective data analysis	700-bed teaching hospital in Eastern India	The CLABSI rates declined by 37.61% and this was matched with an increase in the hand hygiene compliance rates	**Decrease**
Parriott [23]	COVID-19	CLABSI, *C.difficile* infections, MRSA BSI	USA	Retrospective before-and-after study (interrupted time series analysis)	NHSN data from Californian acute hospitals	Substantial and significant increases in the SIRs for CLABSI and MRSA BSI from 2019 to 2020.	**Increase**
Pate *et al.*, 2022 [36]	Presence of central line	CLABSI rates Number of audits completed	USA	Quality improvement project with audit tool and feedback	874-bed, level 1 trauma and academic medical center in Charlotte (NC)	High levels of audit completion resulted in CLABSI reductions However, two peaks in CLABSI rates were associated with higher volumes of COVID hospitalization and decreased audits	**Increase** Audit tool consisted of 10 different components (see text for further information)
Patel PR *et al.*, 2022 [12]	COVID-19	CLABSI rates	USA	Retrospective data analysis	National Healthcare Safety Network (NHSN) database (nearly all US hospitals)	A 28% increase was observed in the national standardized infection ratios (SIRs) and 45% CLABSI increase in the Upper Northeast region	**Increase** ∗ Preliminary data presented. This was the initial paper from the CDC using NHSN data, full paper under Weiner-Lastinger LM below.
Patel SA *et al.*, 2022 [25]	COVID-19	CLABSI cases	USA	Retrospective data analysis	Single hospital (VA network)	Seven CLABSI reported in a 5-months period in a hospital that had experienced none in the 18 months before November 2020	**Increase**
Porto *et al.*, 2022 [28]	COVID-19	HAI incidence, including CLABSI, ventilator associated pneumonias (VAP), proportion of organisms that caused HAI, and antibiotic consumption	Brazil	Retrospective data analysis	21 Brazilian hospitals (Intensive Care Units, ICUs)	Significant increase in median CLABSI incidence during the pandemic Significant increase in the proportion of CLABSI caused by *Enterococcus faecalis* and *Candida spp* during the pandemic	**Increase**
Rosenthal *et al.*, 2022 [31]	COVID-19	HAI, including CLABSI, CAUTI and VAE	India, Mongolia, Jordan, Lebanon, Palestine, Egypt, Turkey	Retrospective data analysis	International Nosocomial Infection Control Consortium (INICC) Surveillance Online System (ICUs in 7 low- and middle-income countries, LMICs, for a total of 7,775 patients)	Increase in CLABSI rates (2.54 versus 4.73 per 1000 central line days) when comparing 2019 (non-COVID) versus 2020 (COVID)	**Increase**
Wang *et al.*, 2022 [21]	COVID-19	CLABSI and blood-culture contamination rate	USA	Retrospective cohort study	University of Texas Medical Branch (UTMB) health system	COVID-19 was associated with significant increase in blood culture contamination and CLABSI rates	**Increase** Black race, end-stage renal disease and obesity as significant risk factors
Weiner-Lastinger *et al.*, 2022 [58]	COVID-19	HAIs, including CLABSI, CAUTI, CDI and MRSA infections	USA	Retrospective daa analysis	National Healthcare Safety Network (NHSN) database (nearly all US hospitals)	Significant increase in the state-level standardized infection ratios (SIRs) for CLABSI observed in 2020	**Increase**

**Table III:** Summary of potential causes for the increase in CLABSI rates, lessons learns and domains that need to be addressed to improve health systems resilience around the globe

**Potential causes for the increase in CLABSI rates**	A. Increased admissions of severely unwell patients B. Increased workload often overwhelmed the personnel, and often redeployment in other unfamiliar units C. Significant reliance on the use of agency staff D. All of the above seem to have led to a reduced compliance with standard IPC measures E. Other factors may also have contributed (i.e., absence of infection specialists, acuity of care, patients' co-morbidities)
**Potential measures to reduce CLABSI rates**	A. Audit tool of IPC practices B. Line care bundle C. Additional capacity of intensive care beds (or the flexibility to increase capacity/availability of trained personnel) D. Economic incentives (as in the case of Israel and CMS)
**Further recommendations**	Healthcare organizations should focus on increasing their compliance rates and on robust auditing processes (in particular, on hand hygiene and line care bundles) Increase local resilience and training of healthcare workers Safeguards in place to preserve and support infection prevention programs during future pandemics (i.e., including financial incentives where feasible) Routine CLABSI surveillance at national level in various healthcare systems Implementation and economic support of national and international public health agencies

### Risk of bias in studies

**Figure 2:**
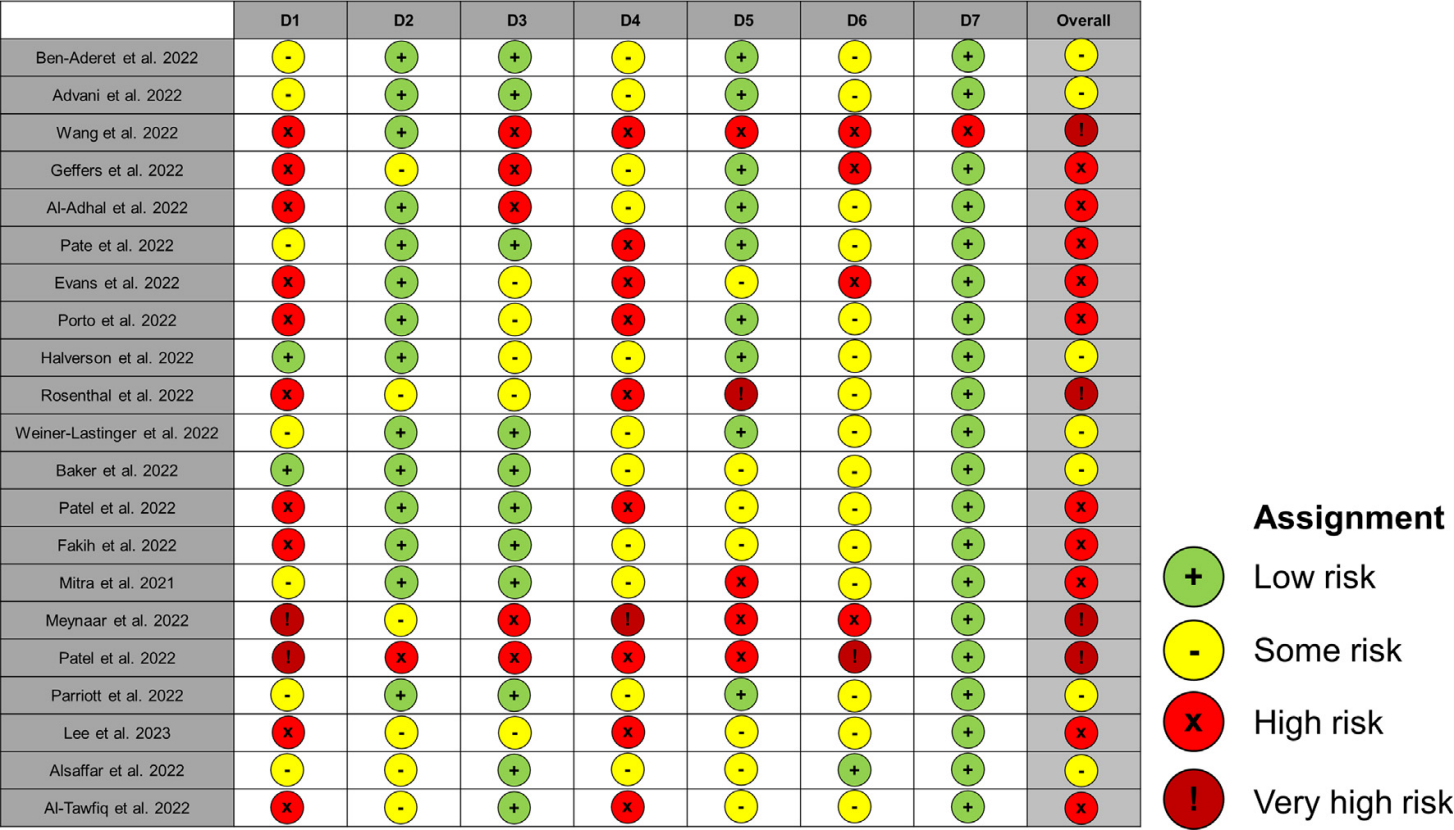
ROBINS-E assessment tool summarising the risk of bias for the studies included within this review assessing different bias domains.

Figure 2 summarises the risk of bias the studies included within this review. Overall, most studies were ranked as high-risk (10/21, 48%) or some-risk (7/21, 33%) of bias. Four (19%) studies were ranked as very high risk of bias. No studies were considered low risk of bias.

#### Was the incidence of CLABSI different during the COVID-19 pandemic?

Overall, most studies analysed (*N*=17) confirmed a significant increase in CLABSI incidence or rates during the COVID-19 pandemic. Only one study (*N*=1) from a single center in India [13] showed a decrease, whilst three other studies (*N*=3), one from Germany [14] and two from Saudi Arabia [15,16], showed no increase and no significant difference when comparing CLABSI rates in pre-pandemic and pandemic periods.

One of the most significant studies on CLABSI rates during COVID-19 comes from the CDC [7]. Virtually all hospitals in USA have mandatory reporting of their CLABSI rates to the NHSN. The analysis of the NHSN database has shown a significantly higher incidence in central line-associated bloodstream infections (CLABSI) and methicillin-resistant *Staphylococcus aureus* (MRSA) bacteraemia in 2021 compared to 2019. These increases generally coincided with periods of high COVID-19 hospitalizations and were especially elevated during the first and third quarters of 2021. The study represents an overall picture of the entire US epidemiology, but further smaller studies have also highlighted similar conclusions. Their preliminary data were also presented in another early study focusing only on the first few months of 2020, demonstrating a 28% increase in the national standardized infection ratios (SIRs) and 45% CLABSI increase in the Upper Northeast region [12].

Another extensive dataset is represented by the Hospital Corporation of America (HCA) network, including 148 hospitals all over USA [17]. It represents the largest health system in the US by number of hospitals affiliated [18] and they reported an increase of 60% in CLABSI rates during the year 2020 of the pandemic. A similar increase was also reported by another major American health system (Ascension) [8]. CLABSI rates increased by 51.0% during the pandemic period, from 0.56 to 0.85 per 1000-line days (*P*<0.001) and by 62.9% from 1.00 to 1.64 per 10,000 patient days (*P<*0.001). Of note, both studies used the same data submitted to the NHSH database for their analyses.

Another retrospective longitudinal multicenter cohort study including 53 hospitals (academic and community) in South-eastern United States [19] also confirmed a significant increase in CLABSI by 24% during the pandemic. In particular, CLABSI rates increased in the later phases of the pandemic and especially in smaller community hospitals, rather than the bigger academic medical centers. The authors postulated that this difference may be due to the lack of infectious diseases expertise in smaller hospitals, but also the fact that later in the pandemic there was a shortage of healthcare staff with redeployment of infection prevention nurses and/or the excessive use of travelling workers, likely causing lapses in infection prevention control (IPC) practices.

Various other studies from Illinois [20], Texas [21], California [22,23] and the Veteran Affairs (VA) system [24,25] also showed a significant increase in CLABSI rates during the pandemic. The data from Illinois not only highlighted a significant increase in CLABSI per 1000 patient days but also a wider increase in the total number of infections per 1000 patient days (*P<*0*.*05). Their analysis also compared staffing levels and demonstrated that there were significant increases in percent of hours that were premium pay (*P<*0*.*005), nurse per patient days (*P<*0*.*0005), agency hours (*P*<0.01), and percent of premium pay that were agency hours (*P*<0.0001), confirming again higher numbers of travelling staff and overstretched healthcare workers during the pandemic [20]. The study from Texas also confirmed a similar association between COVID-19 and an increase in CLABSI rates [21]. The authors also noted an increase in the blood culture contamination rate. Blood cultures should always been taken using an antiseptic technique and an increased rate of contamination is a potential marker of suboptimal IPC measures [26].

The acuity of care may have also contributed to higher CLABSI rates in an academic tertiary-care teaching hospital in Los Angeles [22]. As all other studies, colleagues from California reported their rate of COVID-19 CLABSI as being significantly higher than non–COVID-19 CLABSI. However, these CLABSI occurred predominantly in the intensive care unit (ICU), and the ICU COVID-19 CLABSI rate was significantly higher than the ICU non–COVID-19 CLABSI rate. The prone positioning of COVID-19 patients has been widely used during the pandemic to improve oxygenation. However, the process of turning these patients can cause pulling and friction at the line site and, as patients lay prone for many hours, fluid from the oral cavity can drip toward the line site without a clear visualization from healthcare staff, compromising the dressing integrity and potentially increasing the risk of infection [27].

The acuity of care seemed to have had an impact on the CLABSI rates within the VA system too [24]. During the pandemic, significant increases in the rates of CLABSI and MRSA infections were observed in the VA acute care, but not in the long-term care facilities. This also links with the previous data showing an increase of CLABSI rates in community hospitals [19], highlighting the contrast in the acuity of patients between short-term and long-term healthcare facilities. Patients admitted to the latter have generally less invasive devises and they are generally fewer compared to acute hospitals, potentially leading to better infection prevention and control practices in those settings [24].

The significant increase in CLABSI rates is not limited to the US, but it has also been described at global level. Higher incidences of line infections among COVID-19 patients were observed in Brazil [28], the Netherlands [29], South Korea [30] and some LMICs [31]. The data from 21 Brazilian ICUs showed a significant increase in the median CLABSI incidence during the pandemic. Compared to the pre-pandemic years, they observed an increase in CLABSI incidence during the pandemic in 18 hospitals, whereas 2 hospitals showed a decrease in CLABSI and 1 hospital did not report any CLABSI in either period. Overall, they detected an increase in CLABSI rate during the pandemic of 2.81 (IQR, 1.35–6.89) versus 1.60 (IQR, 0.44–4.20; *P*=0.002 in the pairwise comparison) in the pre-pandemic period.

A study from the Netherlands [29] compared the CLABSI rates among COVID-19 and non-COVID-19 patients admitted during the same time period. They found that the incidence of CLABSI was 1.99/1000-line days in non-COVID-19 patients versus 6.25/1000-line days in COVID-19 patients, confirming the hypothesis that some risk factors may be peculiar only to COVID-19 patients increasing the risk of developing a CLABSI.

In South Korea [30], the rates of BSI and CLABSI significantly increased during the COVID-19 pandemic compared to the pre-COVID-19 period in large-sized hospitals, whereas these rates decreased in small-to-medium-sized hospitals. The reasons for this difference are not apparent and, on further segmented regression analysis, the rate of CLABSI demonstrated an increasing trend during the COVID-19 pandemic even in small-to-medium-sized hospitals, but without reaching a statistical significance.

Data from other LMICs (India, Mongolia, Jordan, Lebanon, Palestine, Egypt and Turkey) using the International Nosocomial Infection Control Consortium Surveillance Online System (including different ICUs for a total of 7775 patients) confirmed an increase in CLABSI rates (2.54 versus 4.73 per 1000 central line days) when comparing 2019 versus 2020 [31]. The issues of high workload, redeployment and overwhelmed healthcare staff during the pandemic, were again recurrent themes at global level, likely to have negatively influenced basic infection control measures and favoured the occurrence of line infections.

In contrast with all the data from around the world, there are three studies that did not show any increase in CLABSI rates. One study in particular [13] represents so far the only published article showing a decrease in CLABSI rates during the COVID-19 pandemic. The authors performed a retrospective analysis in a 700-bed multispecialty teaching hospital in Eastern India, comparting their CAUTI, CLABSI, ventilator-associated pneumonia (VAP), surgical site infections (SSIs) rate pre- and post-pandemic. They also assessed their hand hygiene compliance rates during the same periods. In contrast with all other published studies (including some other from India), their CLABSI rates declined by 37.61% during the pandemic periods and this was matched with an increase in the hand hygiene compliance rates. The latter varied from a minimum of 82% among housekeepers to a maximum of 98.52% among nurses.

A similar study design was also performed in a 500-bed hospital, including 80 adult ICU beds, in Saudi Arabia [15], where the authors assessed the device associated infections (DAIs) and the compliance with hand hygiene in a retrospective observational study comparing pre- and post-pandemic years. There was no significant difference in the number of DAIs or in the compliance with hand hygiene during those different periods. The authors also commented that the strict adherence to IPC measures had probably an impact in reducing the event of DAIs, but their results could not be generalized as limited to a single hospital. This is in line with another Saudi Arabian study from an hospital network with five ICUs [16], where they also recorded no difference in the CLABSI rates, but in contrast with a national analysis including all the Ministry of Health hospitals [32] where the COVID-19 pandemic was associated with increased CLABSI rates.

A wider study was carried out in Germany and it also represents an interesting exception in the global scenario [14]. This interrogated a quite extensive database using the National Reference Center for Surveillance of Nosocomial Infections that includes 921 German ICUs. An epidemiological analysis assessing the incidence of CLABSI and bloodstream infections associated with the use of Extracorporeal-Life-Support-Systems (ECLSABSI) during the pandemic did not find any difference when comparing COVID-19 and non-COVID-19 periods.

#### Were there any other factors associated with the risk of line infection? And what are the most effective preventive measures?

Various publications have previously assessed the risk factors for CLABSI in the pre-COVID era [4,33]. In this literature review, only two studies (out of the 21) have assessed the presence of specific risk factors for CLABSI in COVID-19 patients. In a study from Los Angeles [22], elderly age, diabetes and admission to ICU were all risk factors for CLABSI, as patients with those characteristics had much higher CLABSI rates. Interesting findings from the Netherlands have showed that the risk of CLABSI was significantly increased among COVID-19 patients treated with dexamethasone with or without interleukin antagonists [29].

Other factors, including nursing-related practices, may have contributed to the increased risk of CLABSI. Wearing personal protective equipment (PPE) can be very tiring (and time consuming when donning and doffing) and this may have forced nurses and doctors to batch their tasks when caring for COVID-19 patients. This may have caused substandard IPC practices if rushing through time-critical tasks, such as the disinfection and care of intravascular devices [34]. Redeployment of staff in ICU from non-critical care areas and the use of temporary agencies is also another factor mentioned by some authors [19,34] as these temporary personnel may have had less experience in dealing with high-risk patient and CLABSI prevention practices.

In terms of most effective CLABSI preventive measures, some answers may lie in the studies that have shown a decrease or no difference in line infections rates during the pandemic [[13], [14], [15], [16]]. A high rate of hand hygiene compliance was a common theme, as well as high compliance with line care bundle. Such simple measures are considered the cornerstones of IPC practices but compliance rates can be variable in particular when healthcare staff are under pressure or when wearing extensive PPE for prolonged time.

In a study from Illinois only published as an abstract at the APIC conference [35] at the time of writing this report, the authors have completed a gap analysis where they have identified inconsistent use of the central line insertion checklist and nursing maintenance handoff tool and lack of frontline staff participation in a CLABSI committee as important IPC practices that were partially neglected during the pandemic. Their re-implementation led to a 47.0% decrease in the number of CLABSI. Similar issues were also identified in the VA health system in Nebraska, where they recorded deviations in nursing training, documentation, and standard practices in central-line dressing care, also leading to the omission of the recommended discs impregnated with chlorhexidine gluconate (CHG) [25].

The importance of compliance with IPC practices and its relation with CLABSI rates has also been highlighted in a study conducted in an academic medical center in North Carolina [36]. Compliance with an audit tool with ten different components (type of line, chlorhexidine disk compliance, cleanliness of needleless connectors, presence of alcohol caps, compliance with labelling of tubing, presence of blocked lines, completion of a daily antimicrobial bath, and daily assessment of line necessity) resulted in a CLABSI decline. However, a reduction in the number of audits being completed due to the overload during the coronavirus peaks was linked with two peaks in CLABSI rates, confirming the importance of standard IPC measures.

In a retrospective cohort study from 100 hospitals in Thailand (with at least 200 beds and 10 intensive care unit beds), the authors assessed the reported compliance with IPC measures during the pandemic and they compared the results with a previous identical study in the same units [37]. Comparing 2014 to 2021, there was a reported increase in the following CLABSI preventive measures: chlorhexidine gluconate insertion site antisepsis (73.6% vs 85.0%, *P=0.*03) and maximum sterile barrier precautions (63.2% vs 80.0%, *P=0.*003). However, the lack of CLABSI data and the reporting of self-compliance also represent some major limitations for this study.

It is also interesting to note that during the pandemic many healthcare professionals supported the practice of double-gloving, where two pairs of gloves are worn over each other and the first pair is generally not removed but disinfected with alcohol gel. The aim is to provide further protection to healthcare workers and reduce the number of hand washings using soap and water (as it can dry the skin quite significantly). Some IPC colleagues were worried about the higher risk of outbreaks due to previous reports [38], but a study from Israel showed that double-gloving implemented together with a strict active bacteriological surveillance did not increase the risk of bacterial cross-transmission or CLABSI rates [39]. However, further studies are needed to assess the reproducibility of such findings.

Another study from Israel confirmed high levels of compliance with CLABSI preventive measures during the pandemic but did not contain any data to assess if such compliance had a significant impact on CLABSI rates. Data from 15 different hospitals [40] reported a consistent and full compliance with IPC measures (including sterile barrier precautions and use of chlorhexidine) to prevent line infections during the pandemic. However, the authors did not assess if such high compliance was associated with reduced CLABSI rates, representing a major limitation of the study and preventing any interpretation of their findings. Nevertheless, other authors have shown that lower compliance is generally linked to higher infection rates [36] and the Israeli paper also highlights an interesting factor: the prevention of CLABSI in Israel is incentivized, including monetary compensation from the Department of Health, potentially contributing to the increased compliance with IPC measures.

It is worth noting that the Centers for Medicare & Medicaid Services waived all the mandatory healthcare associated infections (HAIs) reporting requirements during the first peak of the pandemic [34], and the data submitted for the period January–June 2020 were not used for performance calculations, hence not counting for any penalty or incentive programs. This may have had the double effect of potentially reducing the focus on CLABSI surveillance and missing early signals of increased rates.

To summarize the most effective preventive measures in reducing the risk of CLABSI in COVID-19 patients, it seems that strict compliance with IPC practices, in particular hand hygiene and line care bundle, remains as the most cited solution, but there are various challenges (i.e., excessive workload, acuity of patients, redeployment of healthcare staff to mention some) that can significantly hamper such compliance.

The paper from Germany [14], showing no increase of device associated infections for COVID-19 patients admitted in their intensive care units, also highlights some wider considerations to be considered. The authors speculate that the lack of increased healthcare associated infections during the pandemic may be due to two main factors: firstly, Germany has had an overall lower incidence of COVID-19 compared to the US; secondly, Germany has a very high availability of ICU beds compared to all other developed countries [41], making the country much better prepared to deal with the unprecedented surge in severely unwell COVID-19 patients.

#### Was there any difference in the microbial epidemiology?

Different reports have confirmed a change in the traditional microbiological epidemiology of CLABSI during the recent pandemic, with different local variations. In the article from 21 Brazilian ICUs [28], the authors also collected information regarding the microbial pathogens causing infection. A significant increase in the proportion of CLABSI caused by *Enterococcus faecalis* and *Candida* spp. was observed [28]. Similar results were also reported from a community hospital in New York city, where *Enterococcus* spp., *S. aureus* and *Candida* spp. were the more common pathogens identified from bloodstream infections during the COVID-19 surge [42]. Enterococci are a leading cause of healthcare associated infections and they often cause hospital outbreaks, when strict infection control procedures are not implemented to minimize nosocomial spread [43]. Data from both US and European databases (NHSN and Eurobact II) [10,44], confirmed a different epidemiology of bloodstream infections in COVID-19 compared to non-COVID-19 critically ill patients, with increased number of enterococcal infections. Other smaller reports and case series also highlighted an unexpected high incidence of enterococcal bloodstream infection in COVID-19 patients admitted in the intensive care units [45,46], with some authors even postulating a new special pathogen-to-pathogen relationship between SARS-CoV-2 and *Enterococcus* spp. in the human microbiome [47].

The HCA network saw a significant increase in multidrug resistant organisms, including MRSA, vancomycin resistant enterococcus (VRE), and Gram-negative organisms causing bloodstream infections [17]. An increase in the incidence of MRSA infections was also observed statewide in California, in a study analysing the CLABSI rates in the majority of acute hospital facilities in the state during the pandemic [23]. Other hospitals reported an increased in fungal infections and other organisms, but not enterococci. In the Ascension network [8], coagulase-negative staphylococci CLABSI increased by 130% from 0.07 to 0.17 events per 1000-line days (*P<*0.001), and *Candida* spp. by 56.9% from 0.14 to 0.21 per 1000-line days (*P*=0.01). Similar increases in *Candida* spp. and coagulase-negative staphylococci were also observed in some university hospitals in London (UK) [48,49], whilst data from California [22] showed *Candida* spp. were more frequent in COVID-19 CLABSI (45%vs 23%; *P*=0.0150) and other gram-negative organisms in non–COVID-19 CLABSI (27% vs 11%; *P*=0.0337).

Even if not strictly related to CLABSI rates, other authors [50,51] also reported different bacterial outbreaks with unusual or multi-drug resistant pathogens in dedicated COVID-19 wards and intensive care units. This highlights two potential findings: an altered microbial epidemiology among COVID-19 patients and suboptimal IPC measures as already described, favouring the spread of such bacteria.

## Discussion

The main aim of this review was to evaluate extant published data on global CLABSI rates during the recent COVID-19 pandemic; we found most studies describe a statistically significant increase. A secondary aim was also to determine specific risk factors and the most effective preventive measures in reducing the incidence of CLABSI in COVID-19 patients. Unfortunately, much fewer papers have assessed such evidence, but some studies highlight the importance of compliance with IPC practices, in particular hand hygiene and line care bundles. Finally, a tertiary aim was to review the microbiological epidemiology and current evidence details various changes in microbial pathogens causing CLABSI among COVID-19 patients.

Over the last few years, a worldwide effort has been directed to promote hand hygiene in healthcare settings and it is often said that hand hygiene is the single-most effective intervention to reduce hospital acquired infections. However, there is a persistent and recurrent problems in all the different healthcare systems around the world that adequate compliance with hand hygiene cannot be reliably guaranteed [52]. The recent COVID-19 pandemic was no exception. A combination of different factors (i.e., increased workload, acuity of care with very sick patients, overstretched healthcare workers and redeployment) have created extremely challenging circumstances where the compliance may have dropped due to other competing priorities. It is interesting to note that some of the studies reporting increased CLABSI rates have also reported an impact on other HCAI, including catheter-associated urinary tract infections (CAUTI) and ventilator-associated events (VAE), further highlighting the drop in IPC measures [7,17,19,24].

A similar issue was also encountered for the compliance with line care bundle. For the last two decades, a line care bundle (in other words, a set of evidence-based interventions to be implement at the same time to be effective) has provided strong evidence in reducing (up to 66%) the rates of catheter-related bloodstream infections in the intensive care settings [53]. The recommended interventions are hand washing, using full-barrier precautions during the insertion of lines, cleaning the skin with chlorhexidine, avoiding the femoral site whenever possible, and removing unnecessary intravascular catheters. However, these procedures require both experience and time to be implemented and the already mentioned challenging circumstances during the pandemic may have hampered the overall compliance.

There are some important limitations to consider when conducting a review on CLABSI and COVID-19. Most of the studies included in this review are at high-risk of bias. More importantly, selection biases may also be present as only articles highlighting an increase may have been favoured for publications. The ecological analyses looking at the large-scale impact of the pandemic and using the extensive NHSH database [7] did confirm a national increase in the CLABSI rates in US, but it did not include a more detailed analysis. Only countries or hospitals with a well-established surveillance system may have been able to easily collect and publish the data, limiting most of the publications to USA, where CLABSI reporting is mandatory. This also highlights the importance of introducing a mandatory CLABSI reporting, as many healthcare systems (in both developed and developing countries) do not provide such data. In addition, most of the papers only reported the data without providing additional evidence to explain such increase. Only few studies have successfully demonstrated a link with acute and community hospitals, lack of IPC audits, increased workload and use of temporary agency staff [[19], [20], [21],^36^].

Finally, it is important to consider the wider issues at national and global levels. The coronavirus pandemic was an unprecedented event, that has overwhelmed the majority of healthcare systems all over the world. The reality is that most of the countries were ill-prepared to deal with the pandemic and most of the healthcare systems nearly collapsed, requiring a suspension of all non-urgent elective activities and operations. Numerous lessons have been learnt and various domains need to be addressed to improve health systems resilience around the globe [54]. Any solution at improving CLABSI rates during another surge or pandemic will inevitably have to adopt a wider system-thinking approach in addition to more specific local measures (Table III).

## Conflict of interest statement

The authors have no conflict of interest to declare relevant to this manuscript. Outside the work in this manuscript, LSPM declares consulting for/receiving speaker fees from bioMerieux (2013–2023), Eumedica (2016–2022), Pfizer (2018–2023), Umovis Lab (2020–2021), Kent Pharma (2021), Pulmocide (2020–2021), Sumiovant (2021–2023), Shionogi (2021–2023), and received research grants from the National Institute for Health Research (2013–2023), CW+ Charity (2018–2023), and LifeArc (2020–2022).

## Ethics

No ethical approval was required for this review.
